# Smooth muscle SIRT1 reprograms endothelial cells to suppress angiogenesis after ischemia

**DOI:** 10.7150/thno.39320

**Published:** 2020-01-01

**Authors:** Yong-Qing Dou, Peng Kong, Chang-Lin Li, Hong-Xing Sun, Wei-Wei Li, Yuan Yu, Lei Nie, Li-Li Zhao, Sui-Bing Miao, Xiao-Kun Li, Chen Dong, Jin-Wen Zhang, Yang Liu, Xiao-Xia Huo, Kui Chi, Xiang Gao, Ning Zhang, Lin Weng, Hongyuan Yang, Fan Zhang, Mei Han

**Affiliations:** 1 Department of Biochemistry and Molecular Biology, College of Basic Medicine, Key Laboratory of Medical Biotechnology of Hebei Province, Hebei Medical University, Shijiazhuang, China.; 2 Department of Vascular Surgery of Second Hospital, Hebei Medical University, Shijiazhuang, China.; 3 Department of Gastroenterology of Second Hospital, Hebei Medical University, Shijiazhuang, China.; 4 Key Laboratory of Integrative Medicine on Liver-kidney patterns of Hebei Province, College of Integrated Chinese and Western Medicine, Hebei University of Chinese Medicine, Shijiazhuang, China.; 5 School of Biotechnology and Biomolecular Sciences, University of New South Wales, Sydney, Australia.

**Keywords:** cZFP609, angiogenesis, vascular smooth muscle cells, ischemia, exosomes.

## Abstract

**Objective:** Vascular smooth muscle cells (VSMCs) undergo the phenotypic changes from contractile to synthetic state during vascular remodeling after ischemia. SIRT1 protects against stress-induced vascular remodeling via maintaining VSMC differentiated phenotype. However, the effect of smooth muscle SIRT1 on the functions of endothelial cells (ECs) has not been well clarified. Here, we explored the role of smooth muscle SIRT1 in endothelial angiogenesis after ischemia and the underlying mechanisms.

**Methods:** We performed a femoral artery ligation model using VSMC specific human SIRT1 transgenic (*SIRT1*-Tg) and knockout (KO) mice. Angiogenesis was assessed in *in vivo* by quantification of the total number of capillaries, wound healing and matrigel plug assays, and *in vitro* ECs by tube formation, proliferation and migration assays. The interaction of HIF1α with circRNA was examined by using RNA immunoprecipitation, RNA pull-down and *in situ* hybridization assays.

**Results:** The blood flow recovery was significantly attenuated in *SIRT1*-Tg mice, and markedly improved in *SIRT1*-Tg mice treated with SIRT1 inhibitor EX527 and in *SIRT1*-KO mice. The density of capillaries significantly decreased in the ischemic gastrocnemius of *SIRT1-*Tg mice compared with* SIRT1*-KO and WT mice, with reduced expression of VEGFA, which resulted in decreased number of arterioles. We identified that the phenotypic switching of *SIRT1*-Tg VSMCs was attenuated in response to hypoxia, with high levels of contractile proteins and reduced expression of the synthetic markers and NG2, compared with *SIRT1*-KO and WT VSMCs. Mechanistically, *SIRT1*-Tg VSMCs inhibited endothelial angiogenic activity induced by hypoxia via the exosome cZFP609. The cZFP609 was delivered into ECs, and detained HIF1α in the cytoplasm via its interaction with HIF1α, thereby inhibiting VEGFA expression and endothelial angiogenic functions. Meantime, the high cZFP609 expression was observed in the plasma of the patients with atherosclerotic or diabetic lower extremity peripheral artery disease, associated with reduced ankle-brachial index. Knockdown of cZFP609 improved blood flow recovery after hindlimb ischemia in *SIRT1-*Tg mice.

**Conclusions**: Our findings demonstrate that SIRT1 may impair the plasticity of VSMCs. cZFP609 mediates VSMCs to reprogram endothelial functions, and serves as a valuable indicator to assess the prognosis and clinical outcomes of ischemic diseases.

## Introduction

The progressive occlusion in arterial conduits initiates a series of events during atherosclerotic or diabetic lower extremity peripheral artery disease (PAD), including pre-existing collateral arteries into functional conduit vessels proximally and ischemic angiogenesis distally 1. Arteriole formation distally involves endothelial cell (EC) activation, basal membrane degradation, leukocyte invasion, and proliferation of vascular cells 2. Vascular smooth muscle cells (VSMCs) undergo the phenotypic changes from contractile to synthetic state possibly enabling the mobilization, proliferation during arteriogenesis 2, and are capable to differentiate into pericytes to coat around capillaries during angiogenesis 3. The cytokines and growth factors induce EC proliferation, and new capillary formation that occurs by sprouting or by longitudinal splitting (intussusception) of existing vessels 4, 5. The ability of ECs to proliferate and to secrete growth factors, such as VEGF and PDGF, is crucial for the new vascular network development and subsequent arterialization via recruitment of mural cells.

The key factor regulating VEGF production in ischemic tissues is HIF1α. The regulation of HIF1α activity occurs in large part at post-translational modifications, resulting in its stabilization, nuclear translocation, DNA binding activity, and proper transcriptional activity 6, 7. Noncoding RNAs provide novel post-transcriptional/-translation mechanisms of HIF1α regulation, and have been implicated in hypoxia signaling modulation 8, 9. Despite recent advances in the field of angiogenesis, many details of this complex process have not been elucidated so far, especially about the roles (largely ignored) of VSMCs in the progression of angiogenesis after ischemia.

The silent information regulator 1 (SIRT1), a NAD+-dependent histone deacetylase, is highly expressed in the vasculature 10 and is an important modulator of cardiovascular functions in health and disease. Studies indicate that SIRT1 protects against stress-induced vascular remodeling 11, 12, such as neointimal formation 11, aortic stiffness 13, dissection 14 and atherosclerosis in mice 15, 16. The activation of SIRT1 prevents senescence of vascular ECs and VSMCs 17, 18, suggesting a critical role of SIRT1 in vascular homeostasis and vascular diseases. In fact, SIRT1 prevents stress-induced vascular remodeling largely by maintaining the differentiated phenotype of VSMCs. It has been known that SIRT1 controls the angiogenic activity of ECs, and is required for ischemia-induced neovascularization 10. Controversially, other reports have shown that SIRT1-mediated deacetylation inactivates HIF1α in hypoxic mouse tissues 7, and thus has negative effects on tumor growth and angiogenesis 7. Reduced SIRT1 expression may be correlated with enhanced angiogenesis 19, 20. Although there are extensive studies on the vascular protective effects of SIRT1, it is unclear whether continuous or excessive activation of SIRT1 is available for all atherosclerotic vascular diseases, such as atherosclerotic lower extremity PAD. Furthermore, the effect of smooth muscle SIRT1 on the endothelial angiogenic activity has not been well clarified.

In the present study, we performed a femoral artery ligation model using VSMC specific human *SIRT1* transgenic (*SIRT1-*Tg) and *SIRT1* knockout (*SIRT1*-KO) mice, and showed that *SIRT1-*Tg mice displayed delayed blood flow recovery and reduced density of capillaries and arterioles in the ischemic gastrocnemius. cZFP609 was expressed and released by *SIRT1-*Tg VSMCs via exosomes. Furthermore, the cZFP609 reprogramed ECs to suppress angiogenesis via blockade of HIF1α nuclear translocation and VEGFA expression in response to hypoxia. Thus, cZFP609 may act as a novel indicator to assess the prognosis and clinical outcomes of ischemic diseases.

## Materials and Methods

All the data are available in the article and Supplementary Files, or available from the authors upon request.

### Animals and ethics statement

All animal procedures conformed to the Guide for the Care and Use of Laboratory Animals published by the US National Institutes of Health (NIH Publication, 8th Edition, 2011) and were approved by the Institutional Animal Care and Use Committee of Hebei Medical University. VSMC specific *SIRT1*- transgenic (*SIRT1*-Tg) mice 11 and knockout (*SIRT1*-KO) mice 18 were kindly gifted by Dr. De-Pei Liu and Hou-Zao Chen of Chinese Academy of Medical Sciences and Peking Union Medical College, China. All mice were housed in a specific pathogen-free environment under a 12 h /12 h light-dark cycle and fed rodent diet *ad libitum*.

### Hindlimb ischemia model

We used a previously described mice model of unilateral hindlimb ischemia 1. In brief, the mice (male, 12-14 week-old) were anesthetized with a mixture of oxygen and 1.125% isoflurane. Left unilateral femoral artery occlusion was performed by double ligation of the left superficial femoral artery proximal and distal to the deep femoral artery. Animal numbers are stated with the different experimental results. A sham operation was performed on the contralateral right leg. At the same time, for activation or inhibition of SIRT1, the mice were treated with SIRT1 agonist RSV (2 mg/kg/d, J&K) or inhibitor EX527 (2 mg/kg/d, Cayman) by intraperitoneal injection. For intramuscular injection of AAV 21, 3 weeks before *SIRT1*-Tg mice were performed left unilateral femoral artery occlusion, left gastrocnemius muscles were injected with 1×10^12^ vg /mL AAV9-shRNA-cZFP609 (adeno-associated virus-9 short-hairpin RNA; shcZFP609, HANBIO) and AAV9-shRNA-NC (shNC, HANBIO) by multi-point injection, the injection volume was 10 μL per point, 5 points in total. On day 14 after surgery, mice were euthanized by injection of pentobarbital (80 mg/kg IP) 22, the gastrocnemius tissues were harvested and placed in FSC 22 Frozen Section Media (Leica, 3801480), and three random 10 μm frozen sections of the gastrocnemius muscle per animal were used for immunofluorescence analyses.

### Laser Doppler Perfusion Imaging

For laser Doppler perfusion imaging, the animals were anesthetized and measured under a Periscan PSI imager (Perimed, Sweden). The readings of 3 individual measurements per mouse were averaged for each time point. Measurements were performed just before and after the operation on day 0, 3, 7 and 14 with the last measurement constituting the main end point. Flow ratios of the left / right leg were compared between the treatment groups.

### Human blood sample collection

Human blood sample collection was approved by the Human Ethics Committee, Hebei Medical University Second Hospital and the study was conducted in accordance with the principles of Good Clinical Practice and the Declaration of Helsinki. Written consent from participants or their guardians was obtained. Plasma samples were collected from the initial admission blood draws of those patients who attended the Department of Vascular surgery. Eligible patients were with lower extremity PAD, which were enrolled with an abnormal ankle-brachial index (ABI)≤0.80 at screening. Prior to blood collection, we carefully examined the medical history of control subjects and lower extremity PAD, which having one or more of the following criteria was excluded: prior surgery or trauma within one month prior to admission; autoimmune disease; severe infection; and malignancy. The present study recruited 19 patients with lower extremity PAD and 30 control subjects.

### Wound healing assay

The male *SIRT1*-Tg, *SIRT1*-KO or WT mice (12-14 week-old) were anesthetized with a mixture of oxygen and 1.125% isoflurane. Dermal wound healing assays were done by creating in the back skin of 6 mm punch wounds without injuring the underlying muscle. Wound regions were photographed using a NIKON camera (D 7000) on day 0, 1, 3, 5, and 7. Wound area was calculated using Image J software. Wound sizes at different time points were expressed as percentage of the wound area on day 0.

### *In vivo* Matrigel plug assay

Growth factor-reduced Matrigel (0.5 mL, Fisher Scientific) with or without VEGFA (100 ng/mL, Cell Signaling Technology) were injected subcutaneously into opposite iliac regions of *SIRT1*-Tg, *SIRT1*-KO or WT mice. After injection for 7 d, matrigel plugs were removed from the mice under anesthetization and used to EC invasion assay. Quantification of blood vessels was performed on three sections per plug to define CD31.

### Immunofluorescence analysis

Immunofluorescence staining was performed on acetone-fixed EC monolayers or 10-μm-thick frozen sections. Sections were blocked using 5% normal goat serum in TBS for 30 min and then incubated with primary antibodies against CD31 (BD Biosciences), HIF1α (GeneTex) or α-SMA (Abcam) at 4 °C overnight, and isotype matched controls. Sections were washed 3 times with TBS and incubated with fluorescein-conjugated secondary antibodies (Alexa Fluor® 555 or Alexa Fluor 488, Invitrogen) for 1 h at room temperature. Nuclei were detected by DAPI (Antifade Mountant with DAPI, Thermofisher). Images were acquired using a fluorescence microscope (Olympus, Japan) or a Confocal Laser Scanning Microscope Systems (Leica). Digitized images were analyzed with Image J or software program LAS AF Lite. The total number of capillaries labeled with CD31 was counted on 5 random optical fields for each section. The relative tubule length and number of branches were expressed in capillaries per square millimeters.

### Plasmid construction

To construct cZFP609 expression plasmids, mouse cZFP609 cDNA was synthesized by Sangon Biotech (Shanghai, China) and cloned into pcD-ciR vector (Geneseed Biotech, Guangzhou, China). The pcD-ciR vector contained a front circular frame and a back circular frame. The HIF1α luciferase reporter vector contained two tandem repeats of HIF1α binding site (ACGTG) of the mouse *PDGFB* gene, was inserted into pGL3-Promoter vector (Promega). Transfection was carried out using Lipofectamine 2000 (Invitrogen) according to the manufacturer's instructions.

### Cell culture and treatment

The VSMCs of WT, *SIRT1*-Tg and *SIRT1*-KO mice were isolated from aortas with 1% collagenase, and cultured in low glucose Dulbecco's modified Eagle's medium (DMEM, Invitrogen) supplemented with 10% fetal bovine serum (FBS, Gibco). Passage 4 to 10 cells were used in the experiments. The primary ECs of mice were isolated from the lungs of 4-week-old C57BL/6J mice by two rounds of immunoselection with CD31 (BD Biosciences) and CD102 (BD Biosciences)-conjugated magnetic beads according to a previously described procedure 23, and cultured in Endothelial Cell Medium (ECM, Sciencell) containing 5% FBS and endothelial cell growth supplements (ECGS). Passage 5 to 10 cells were used in the experiments. Human umbilical vein endothelial cells (HUVECs) were obtained from Sciencell and cultured on gelatin-coated plates in ECM supplemented with 5% FBS and ECGS, and used to evaluate endothelial angiogenic functions. Before hypoxia, all of the above cells were incubated in serum-free medium for 24 h, and then under hypoxic (1% O_2_) or normoxic (21% O_2_) conditions for another 24 h. To block the degradation of HIF1α, the cells were pretreated with DMOG (1 mM, Sigma) for 4 h before the collection of cells. For activation or inhibition of SIRT1, the cells were pretreated with resveratrol (RSV, 25 μM, J&K) or EX527 (20 μM, Cayman) for 4 h before hypoxia. To examine the effect of *SIRT1*-Tg VSMCs on EC function, the ECs were incubated with the hypoxia-induced VSMC conditioned culture media for 24 h.

### Cell proliferation and migration assays

ECs or HUVECs proliferation assay was performed as described in the product (2750, Millipore). The cells were labeled for 12 h with BrdU. OD readings were done at 450 nm.

The migration of ECs or HUVECs was evaluated by performing a cell-wounding assay. Cells grown to 100% confluence on glass slides were scraped off the slides with a cell scraper to create a 3-mm-wide wound and were then incubated at 37 ℃ for 24 h under hypoxia. The cells were fixed with methanol and stained with hexamethylpararosaniline. The migration activity of the cells was expressed as the number of cells that migrated into the wound area in each field.

### *In vitro* tube formation assay

Cells were starved overnight in 0.5% FBS, and then detached with trypsin, seeded at a density of 30000 cells per well in a 48-well plate containing reduced-growth factor matrigel (BD Biosciences). Cells were in the conditioned culture media or in media containing 0.5% FBS, VEGFA (100 ng/mL). Tube length was quantified after 24 h by measuring the cumulative tube length in five random microscopic fields. The mean value of 10 cumulative total lengths per well represents an experimental point. The relative tubule length and number of branches were assessed using Image-Pro Plus.

### Exosome Purification

The VSMCs were incubated in serum-free low glucose DMEM for 24 h. The conditioned medium was harvested, and centrifuged at 3,000 g for 15 min at 4℃. The supernatants were then passed through a 0.22 μm (Millipore) filter to remove cellular debris and large vesicles. The clarified medium was mixed with GS^TM^ solution (Geneseed Biotech) and incubated overnight at 4℃, and then centrifuged twice at 5,000 g. The pelleted exosomes were resuspended in approximately 100 μL of PBS and subjected to subsequent experiments.

### Small interfering RNA (siRNA) transfection

The siRNA duplexes targeting mouse cZFP609 (si-cZFP609), 5'- GUCUGAAAAGCAAUGAUGUTT-3' and 5'- ACAUCAUUGCUUUUCAGACTT-3' were obtained from GenePharma. Scrambled siRNA (si-Con) 5'-UUCUCCGAACGUGUCACGUTT-3' and 5'-ACGUGACACGUUCGGAGAATT-3' served as a negative control. The siRNAs were transiently transfected into VSMCs using Lipofectamine® RNAiMAX Transfection Reagent (Invitrogen) according to the manufacturer's protocol.

### RNA isolation and quantitative reverse transcription-PCR (qRT-PCR)

Total RNAs from cell lysates were isolated using TRIzol reagent (Life Technologies). The nuclear and cytoplasmic fractions were extracted using Minute TM Cytoplasmic and Nuclear Extraction Kit (Invent Biotechnologies). To quantify the amount of mRNA and circRNA, cDNAs were synthesized using the M-MLV First Strand Kit (Life Technologies), and quantitative PCRs were performed using SYBR Green qPCR SuperMix-UDG (Life Technologies). For quantification, all RNA expression was normalized to the amount of Tubulin using the 2^-ΔΔCt^ method.

### Western blot analysis

RIPA buffer was used to lyse cells (50 mM Tris-Cl, pH 7.5, 1% NP-40, 0.5% Na-deoxycholate, 150 mM NaCl supplemented with complete proteinase inhibitor, Roche Applied Sciences) and mice muscles (50 mM Tris-Cl, pH 7.5, 1% NP-40, 0.5% Na-deoxycholate, 0.1% SDS, 1 mM EDTA, 150 mM NaCl supplemented with complete proteinase inhibitor). Equal amounts of protein (30~60 μg) were separated by 10% SDS-PAGE, and electrotransfered to a PVDF membrane. Membranes were blocked with 5% milk in TBS for 1 h at room temperature, and incubated with primary antibodies against HIF1α (GeneTex), VEGFA (Arigo), β-actin (Cell Signaling Technology), Lamin A/C (Cell Signaling Technology), Tubulin (Cell Signaling Technology), α-SMA, SM22α, MMP2, MMP9, OPN and NG2 at 4℃ overnight, and then with the HRP-conjugated secondary antibody (Abcam) for 1 h. The blots were evaluated with GE ImageQuant™ LAS 4000 detection system. The protein bands of interest were quantified using Image Pro Plus 6.0 software, and the integrated signal densities were normalized to β-actin or Tubulin (the loading control).

### Fluorescence* in situ* hybridization (FISH)

The ECs were washed in PBS and fixed in 4% paraformaldehyde for 10 min and permeabilized overnight in 70% ethanol. Then the cells were rehydrated for 10 min in 50% formamide and 2×SSC. In case of immunofluorescence, cells were blocked with 10% BSA in PBS for 2 h followed by incubation with HIF1α (GeneTex) (1:200) in PBS treated with DEPC (VETEC) at 4 ℃ overnight. After washing three times in PBS, cells were incubated with secondary antibody (Alexa Fluor 488, Invitrogen).

For FISH, the cells were incubated using specific probes of cZFP609. Hybridization was performed using fluorescence-labeled probes in hybridization buffer by incubation at 55 ℃ for 1 h. After stringent washing with SSC buffer, cell nuclei were counterstained with DAPI (Invitrogen). Images were acquired using a Confocal Laser Scanning Microscope Systems (Leica).

### RNA immunoprecipitationn assay (RIP)

The ECs were washed in ice-cold PBS, lysed in lysis buffer (20 mM Tris-HCl, pH 7.0, 150 mM NaCl, 0.5% NP-40, 5 mM EDTA, with freshly added 1 mM DTT, 1 mM PMSF, and 2 U/μL RNase inhibitor), and then incubated with 5 μg the primary antibody at 4 ℃ for 2 h. 50 μL Dynabeads Protein G (Life technology) was added to each sample, and the mixtures were incubated at 4 ℃ for 4 h. The pellets were washed with PBS and resuspended in 1 mL TRizol Reagent (Invitrogen). The precipitated RNA in the aqueous solution was subject to qRT-PCR analysis to demonstrate the presence of the binding products using respective primers 24.

### RNA pull-down assay

The ECs were washed in ice-cold phosphate-buffered saline, lysed in 500 μL lysis buffer (20 mM Tris-HCl, pH 7.0, 150 mM NaCl, 0.5% NP-40, 5 mM EDTA, with freshly added 1 mM DTT, 1 mM PMSF, and 2 U/μL RNase inhibitor), and then incubated with 3 μg biotinylated DNA oligo probes against endogenous or ectopically expressed cZFP609 at 4 ℃ for 2 h. A total of 50 μL Dynabeads™ MyOne™ Streptavidin C1 magnetic beads (Invitrogen) were added to each binding reaction and further incubated at 4 ℃ for 2 h. The beads were washed briefly with lysis buffer for three times. The bound proteins in the pull-down materials were analyzed by western blot 25.

### Chromatin immunoprecipitation assay (ChIP)

The VSMCs or ECs were incubated at 20% or 1% O_2_ for 24 h and were fixed in 1% formaldehyde for 10 min to cross link proteins with DNA. The cross-linked chromatin was then prepared and sonicated to an average size of 400-600 bp. The samples were precleared with Dynabeads Protein G (Life technology) for 30 min at 4 ℃. The DNA fragments were immunoprecipitated overnight at 4 ℃ with the HIF1α (GeneTex) and normal rabbit IgG (Santa Cruz) antibodies. The precipitated DNA was recovered via phenol/chloroform extraction, and the HIF1α binding site (HBS) was amplified by qPCR. Each experiment was replicated at least three times.

### Statistics

All statistical analyses were performed with the SPSS 21.0 software. The data are presented as means ± SEM. Two groups were compared by Student's T tests. Differences among groups were analyzed with one-way analysis of variance (ANOVA). Blood flow recovery imaging of the mouse foot and wound healing assay were analyzed with ANOVA of repeated measurement data. Spearman rank correlation test was performed to determine the relationship between human plasma cZNF609 level and ABI. Data were analyzed using IBM SPSS Statistics 21.0 (IBM Corporation, Armonk, NY, USA). For all statistical comparisons, *P* < 0.05 was considered significant.

## Results

### Blood flow recovery is delayed after hindlimb ischemia in *SIRT1-Tg* mice

**Figure 1:**
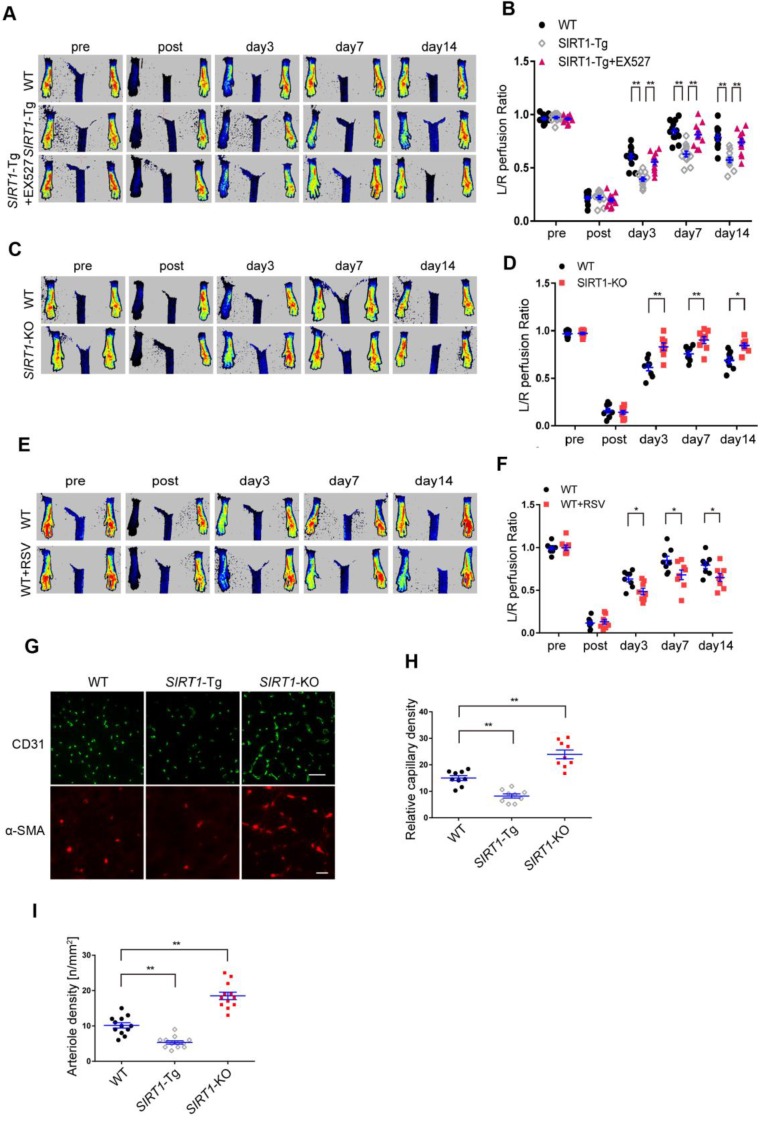
** Blood flow recovery is delayed after hindlimb ischemia in *SIRT1*-Tg mice.** (A-F) Representative laser Doppler perfusion images at indicated time point after hindlimb ischemia. (A and B) WT, *SIRT1-*Tg and *SIRT1-*Tg mice treated with SIRTl inhibitor EX527 (n= 12). (C and D) WT and *SIRT1-*KO mice (n= 8 per group). (E and F) WT mice treated with DMSO or SIRTl agonist RSV after hindlimb ischemia (n= 8 per group), Laser Doppler perfusion at various time points was expressed as a ratio of flow between ischemic (L) and sham (R) limbs (L/R). (G) Representative immunofluorescence for CD31 (Scale bars = 100 μm) and α-SMA-positive cells (Scale bars = 20 μm) in the gastrocnemius tissue. (H) Immunostaining of CD31-positive cells represented the relative capillary density. (I) The density of arteriole was expressed as the quantity of arterioles per mm^2^. Data represent mean±SEM. Repeated Measures ANOVA, one-way ANOVA or student's t-test:*P<0.05, **P<0.01 versus the corresponding control.

To validate the effects of smooth muscle SIRT1 on angiogenesis 7, 10, 26, we first performed the hindlimb ischemia on VSMC specific *SIRT1-*Tg, *SIRT1-*KO and wild type (WT) mice by ligation of the left femoral artery. Using a laser Doppler blood flowmeter, we found that the blood flow recovery was significantly attenuated in *SIRT1*-Tg mice (Figure 1A-B). Conversely, the blood flow recovery was markedly improved, which displayed increased blood flow perfusion on day 3 after ischemia in *SIRT1-*KO mice (Figure. 1C-D). To determine the causal relationship between SIRT1 and blood flow perfusion, we examined the effect of the activation or inhibition of SIRT1 on blood flow recovery after ischemia. We showed that delayed blood flow recovery was markedly reversed in *SIRT1*-Tg mice treated with SIRT1 inhibitor EX527 compared with untreated control (Figure 1A-B). Conversely, SIRT1 agonist resveratrol (RSV) resulted in a reduced blood flow perfusion in the ischemic hindlimbs of WT mice, similar to *SIRT1*-Tg mice (Figure 1E-F). New capillary formation is an essential prerequisite for arteriole formation in ischemia distally. Then, we examined the capillary density in the ischemic gastrocnemius using immunofluorescence staining of the frozen tissue sections. We showed that the number of capillaries with CD31 positive ECs reduced in ischemic* SIRT1-*Tg tissues, accompanied by decrease in arteriole density (Figure 1G-I). In contrast, increased density of capillaries and arterioles was observed in the ischemic gastrocnemius of* SIRT1-*KO mice (Figure 1G-I). We speculated that the angiogenic dysfunction resulted in delayed blood flow recovery after ischemia in *SIRT1-*Tg mice.

### Angiogenesis is impaired in *SIRT1*-Tg mice

**Figure 2:**
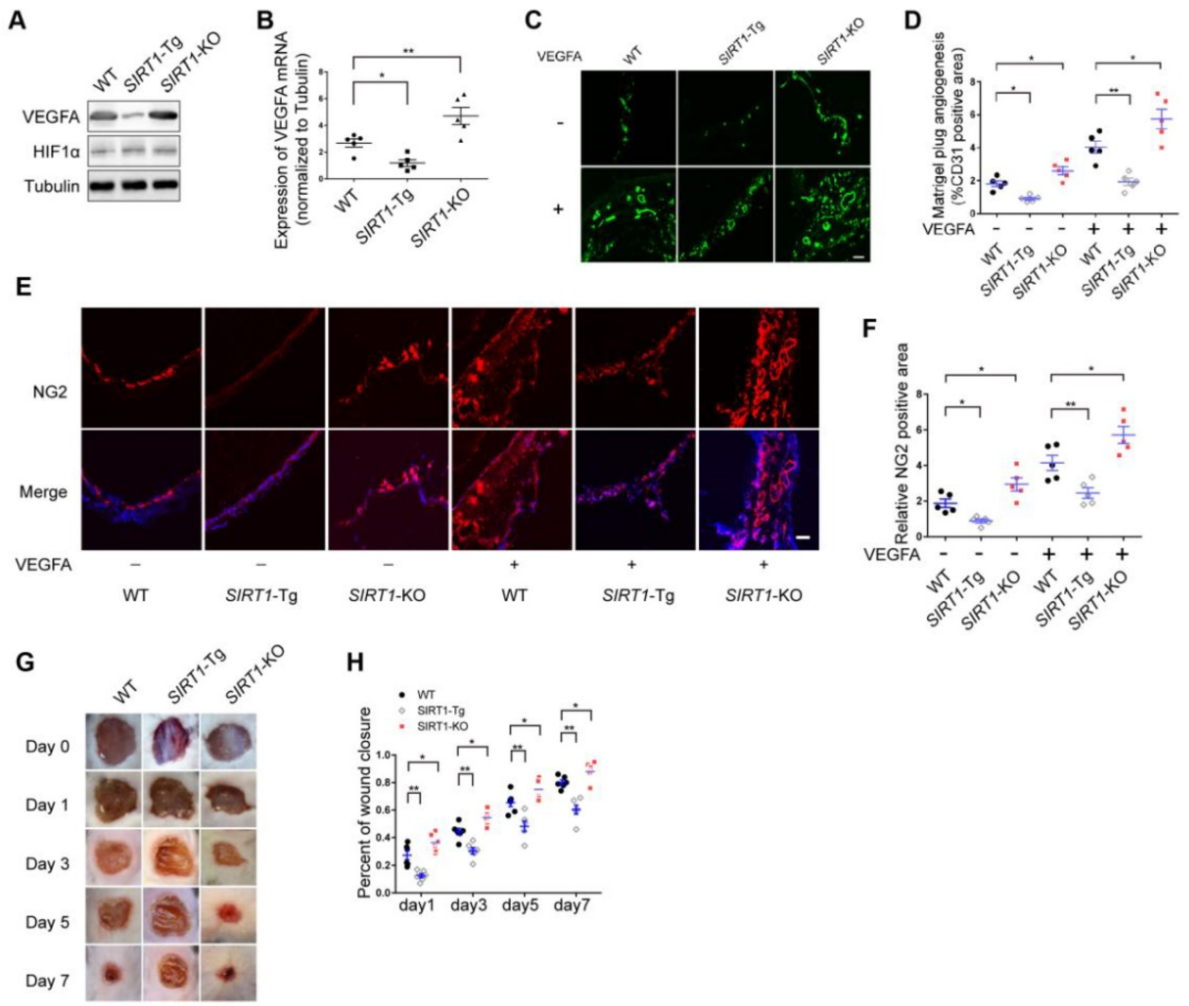
** Angiogenesis is impaired in *SIRT1-*Tg mice.** (A) Western blot of VEGFA, HIF1α and (B) qRT-PCR for the expression of VEGFA in gastrocnemius tissues after 14 days of hindlimb ischemia (n=5). (C) Representative images of CD31 immunostaining in Matrigel plugs containing VEGFA or not implanted in mice (n=5 mice per group). (D) Quantification of angiogenesis was expressed as CD31-positive area for each section in randomly acquired images. Scale bars = 100 μm. (E) Representative images of NG2 immunostaining of Matrigel plugs containing VEGFA or not implanted in mice (n=5 mice per group). Nuclei were stained with DAPI in blue. (F) Relative NG2 positive areas show the number of vessels. Scale bars = 100 μm. (G) Representative images of wound healing at various time points. (H) Wound closure at different time points were expressed as percentage of the wound area on day 0 (n=5 mice per group). Data represent mean±SEM. Student's t-test, one-way ANOVA or repeated Measures ANOVA: *P<0.05, **P<0.01 versus the corresponding control.

Arteriole formation depends on angiogenesis in ischemic distal region via mural cell and SMC coverage 3. Angiogenesis is largely driven by VEGFA production in response to hypoxia 27. To verify the angiogenic dysfunction in *SIRT1*-Tg mice, we first detected the expression of VEGFA in the ischemic gastrocnemius of *SIRT1-*Tg mice using Western blot and qRT-PCR. We showed decreased protein and mRNA level of VEGFA in *SIRT1-*Tg ischemic tissues (Figure 2A-B). Furthermore, this decrease was eliminated in *SIRT1-*KO mice under the same conditions. However, the expression of HIF1α was no difference in the ischemic tissues among WT, *SIRT1*-Tg and *SIRT1-*KO mice. To further confirm the inhibitory effect of smooth muscle SIRT1 on angiogenesis, we performed a matrigel plug implantation assay to evaluate angiogenesis* in vivo*. We found that the number of the capillaries and mural cells invaded into the matrigel with or without VEGFA was reduced in *SIRT1-*Tg mice, and increased in* SIRT1-*KO mice (Figure 2C-F). The inhibitory effect of smooth muscle SIRT1 on angiogenesis was also verified by a puncture wound model in *SIRT1-*Tg and -KO mice, which revealed a delayed wound closure in *SIRT1-*Tg mice (Figure 2G and H). These findings imply that VSMC SIRT1 may disturb angiogenesis and mural cell activation.

### The exosomes of *SIRT1-*Tg VSMCs inhibit the endothelial angiogenic function *in vitro*

**Figure 3:**
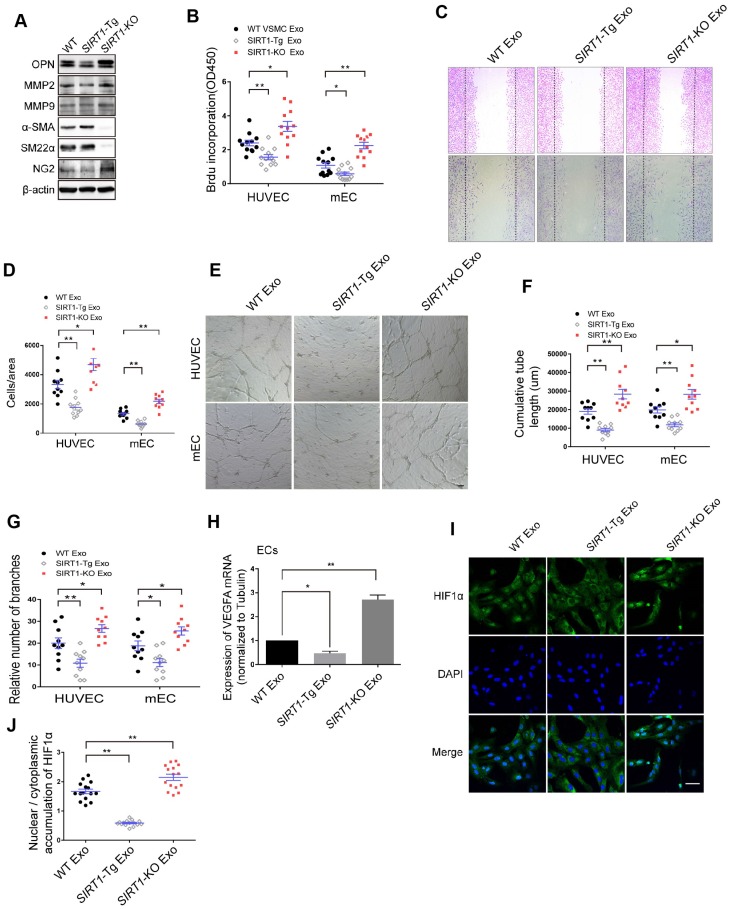
** The exosomes of *SIRT1-*Tg VSMCs inhibit the endothelial angiogenic functions *in vitro*.** (A) VSMCs from WT,* SIRT1*-Tg or *SIRT1*-KO mice were incubated under hypoxia for 24 h. Western blot of OPN, MMP2, MMP9, α-SMA, SM22α and NG2. (B-J) HUVECs or mouse ECs were incubated with the hypoxia-induced VSMC exosomes (Exo) for 24 h and exposed to hypoxia. (B) The relative activity of proliferation by BrdU incorporation. (C and D) The relative activity of migration using a cell-wounding assay. (E) Representative images of tube formation. Scale bars = 200 μm. Relative tube length (F) and number of branches (G) were quantified by measuring the cumulative tube length and branches. (H) qRT-PCR of VEGFA expression in mouse ECs. (I) Immunofluorescent confocal microscopy of HIF1α nuclear translocation in the ECs. Scale bars =100 μm. (J) The quantification of the nuclear-to-cytosol ratio of HIF1α protein in ECs (n=15). Bar graphs show mean±SEM. Student's t-test or one-way ANOVA: *P<0.05, **P<0.01 versus the corresponding control.

To examine the phenotypic state of *SIRT1-*Tg VSMCs in response to hypoxia, we first detected the expression of the phenotypic markers in the cells. We showed that there were lower expression of the synthetic markers OPN, MMP2 and MMP9, and higher level of contractile markers α-SMA and SM22α in* SIRT1-*Tg VSMCs exposed to hypoxia, compared with WT and *SIRT1-*KO VSMCs that displayed increased expression of synthetic markers (Figure 3A), suggesting that* SIRT1-*Tg VSMCs were a differentiated phenotype even under hypoxic conditions. Importantly, the expression of NG2 almost disappeared in *SIRT1-*Tg VSMCs exposed to hypoxia, implying that the plasticity of the VSMCs was impaired to some extent, which might be associated with reduced mural cell coverage. Cells are able to release exosomes into their environment and thereby have an additional form of communication to influence target cell behavior over long distances 28. To find out how *SIRT1-*Tg VSMCs lead to angiogenic dysfunction observed in the model, we isolated the exosomes from the conditioned media of WT, *SIRT1-*Tg and *SIRT1-*KO VSMCs to treat human and mouse ECs, respectively. Using BrdU incorporation, we showed that the exosomes from *SIRT1-*Tg VSMCs significantly reduced the proliferation activity of the ECs from the two species in response to hypoxia (Figure 3B), consistent with the results observed in the ischemic gastrocnemius of *SIRT1*-Tg mice. Migration of ECs is of central importance to angiogenesis. We performed a cell-wounding assay, and showed that the migration of the two ECs was attenuated following incubation with the *SIRT1-*Tg exosomes compared with WT control (Figure 3C-D). However, the exosomes of *SIRT1-*KO VSMCs increased the migration activity of the ECs. Using an *in vitro* tube formation assay, we found that relative tube length and number of branches were significantly reduced in *SIRT1-*Tg exosome group and increased in the cells treated with* SIRT1-*KO exosomes, compared with WT control (Figure 3E-G). The ability of ECs to secrete growth factors, such as VEGF, is crucial for the new vascular network development via induction of proliferation and migration. We performed qRT-PCR of VEGFA, and showed decreased VEGFA expression in ECs treated by the exosomes of *SIRT1-*Tg VSMCs in response to hypoxia (Figure 3H), consistent with the results observed in the model. In addition, this reduction was reversed in ECs treated by the exosomes of *SIRT1-*KO VSMCs, in which the level of VEGFA mRNA was 1.9-fold higher than that of WT control (Figure 3H).

HIF1α nuclear translocation is a key step for activating expression of the target genes including *VEGFA*. We examined hypoxia-induced HIF1α nuclear translocation in ECs treated with the exosomes of WT, *SIRT1-*Tg and *SIRT1-*KO VSMCs using immunofluorescence staining by anti-HIF1α antibody. We found an almost disappeared nuclear HIF1α expression in ECs treated with the exosomes of *SIRT1-*Tg VSMCs under hypoxia (Figure 3I). In contrast, HIF1α was mainly accumulated in the nucleus of ECs treated with exosomes of *SIRT1-*KO cells (Figure 3I-J). These findings imply that some components of *SIRT1-*Tg exosomes may be delivered into ECs and inhibit VEGFA expression via sequestrating HIF1α in the cytoplasm.

### Smooth muscle exosome cZFP609 attenuates hypoxia-induced VEGFA expression in ECs

**Figure 4:**
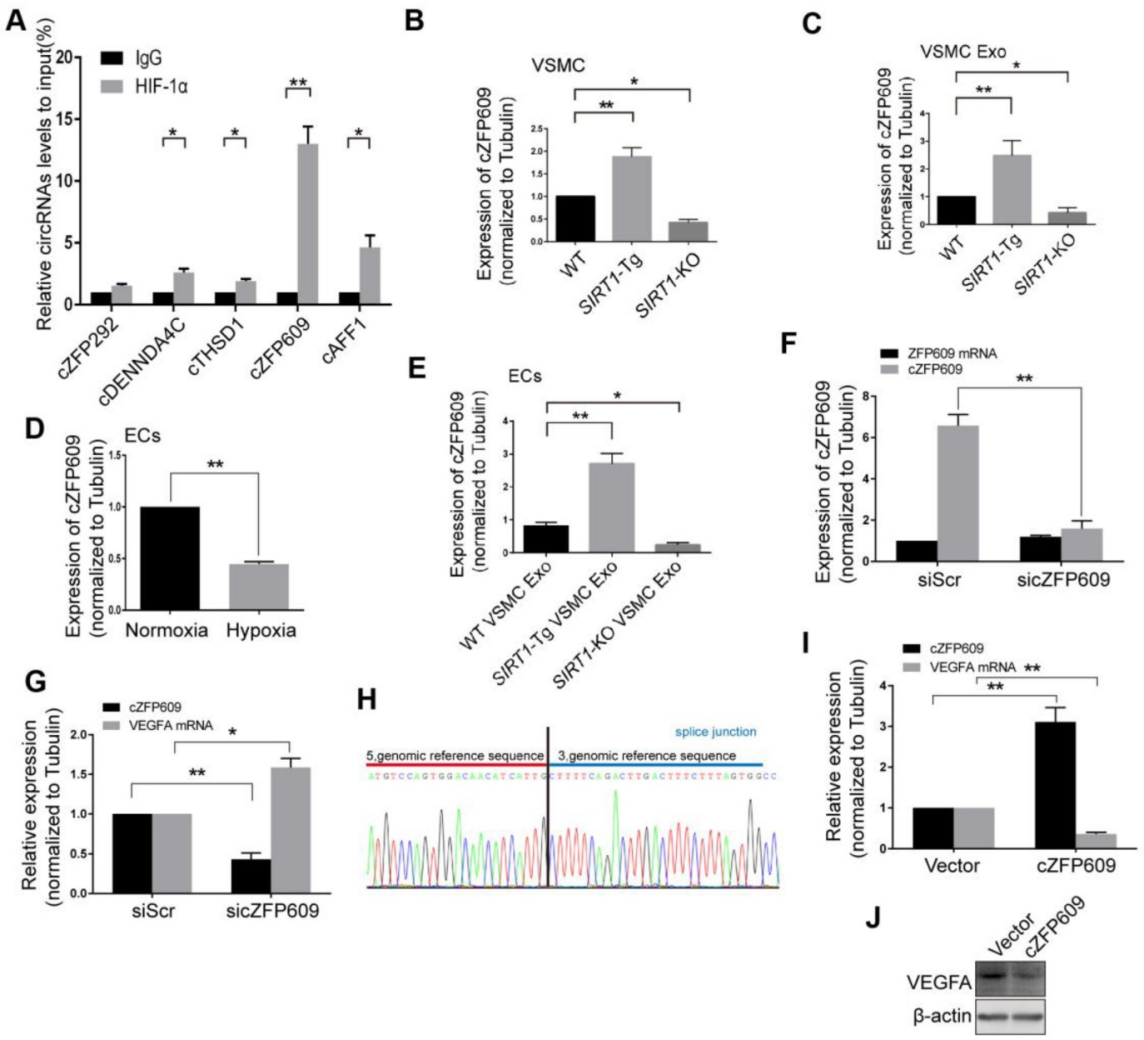
** Smooth muscle exosome cZFP609 attenuates hypoxia-induced VEGFA expression in ECs.** (A-G and I) qRT-PCR. VSMCs or ECs were exposed to hypoxia for 24 h, except if stated otherwise. (A) RIP assay were performed using HIF1α antibodies in ECs treated with the exosomes of *SIRT1-*Tg VSMCs under hypoxia. (B and C) cZFP609 expression in VSMCs (B) and the matched exosomes (C). (D) cZFP609 expression in the ECs. (E) cZFP609 expression in the ECs incubated in the VSMC exosome. (F) cZFP609 expression in *SIRT1*-Tg VSMCs transfected with siScr or sicZFP609. (G) cZFP609 and VEGFA mRNA expression in the ECs treated with the exosome from the cZFP609 siRNA-transfected *SIRT1-*Tg VSMCs. (H) The sequence of cZFP609 was obtained from Sanger sequencing. (I) cZFP609 and VEGFA mRNA expression. The ECs were transfected with vector and cZFP609 for 24 h. VEGFA protein expression were detected by Western blot (J). (A-G and I) Bar graphs show mean±SEM from 3 independent experiments (n=3). Student's t-test or one-way ANOVA: *P<0.05, **P<0.01 versus the corresponding control.

Non-coding RNAs carried by exosomes can be functionally transferred to recipient cells and subsequently regulate gene expression 29. The latest study leads to the identification of 7770 circRNAs in human and mouse VSMCs that shared similar circRNA signatures 30. To determine whether reduced nuclear HIF1α expression in ECs is mediated by circRNAs of *SIRT1*-Tg exosomes, we screened a set of the latest reported circRNAs involved in angiogenesis or regulated by hypoxia as candidate sponge RNAs and assessed their potentially interaction with HIF1α by RIP assay using anti-HIF1α antibody 31, 32. We showed that HIF1α markedly recruited cZFP609 in ECs treated with the exosomes of *SIRT1*-Tg VSMCs under hypoxia (Figure 4A). Furthermore, the exosomes of *SIRT1-*Tg VSMCs knocked down for cZFP609 increased the HIF1α nuclear translocation induced by hypoxia in ECs (Figure S1).The level of cZFP609 expression was higher in *SIRT1*-Tg VSMCs and especially its exosomes under hypoxia (Figure 4B-C). Conversely, *SIRT1*-KO and WT VSMCs displayed lower expression of cZFP609 under the same conditions. However, the level of cZFP609 was decreased in ECs under hypoxia compared with normoxia state (Figure 4D), and significantly increased following incubation with the exosomes from *SIRT1*-Tg VSMCs exposed to hypoxia (Figure 4E). Moreover, this increase was abolished in ECs treated with the exosomes from the cZFP609 specific siRNA-treated *SIRT1*-Tg VSMCs, accompanied by an increased expression of VEGFA with unchanged ZFP609 mRNA (Figure 4F-G), suggesting that cZFP609 is generated by *SIRT1*-Tg VSMCs and delivered to ECs.

To further confirm the inhibitory effect of cZFP609 on VEGFA expression, we construct cZFP609 expression plasmid. Mouse cZFP609 cDNA was synthesized and cloned into pcD-ciR vector (Figure 4H). We showed that overexpression of cZFP609 resulted in reduced expression of VEGFA in ECs compared with vehicle control (Figure 4I-J). These data indicate that the exosome cZFP609 of *SIRT1*-Tg VSMCs attenuates hypoxia-induced VEGFA expression in ECs.

### Knockdown of cZFP609 improves endothelial angiogenic functions

**Figure 5:**
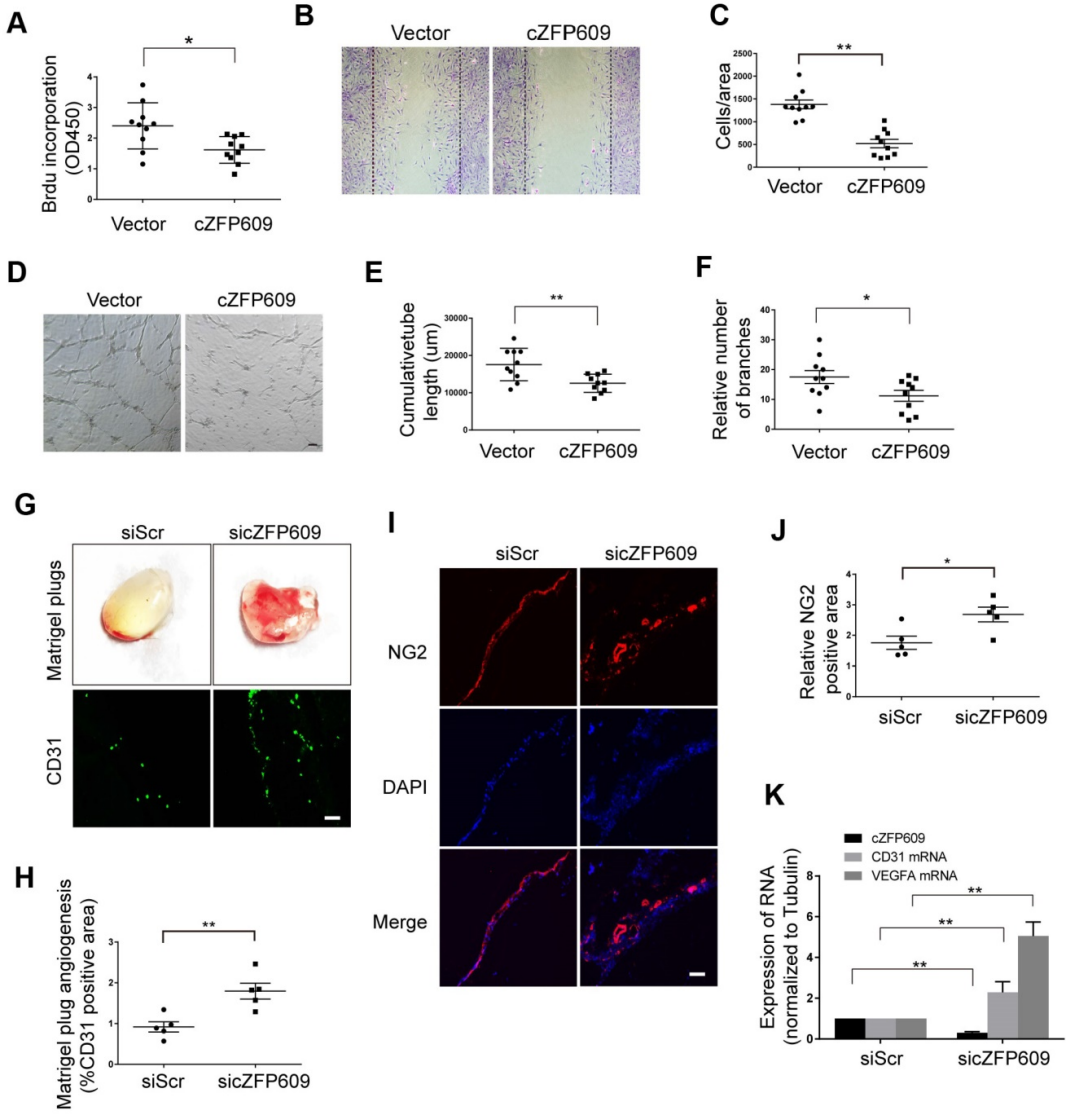
** cZFP609 inhibits the endothelial angiogenic functions.** (A-F) The mouse ECs were transfected with vector or cZFP609 for 24 h, and then treated by hypoxia for 24 h. (A) Proliferation of ECs was determined by BrdU incorporation. Bar graphs show the relative activity of proliferation. (B and C) The migration of cells was evaluated by a cell-wounding assay. The number of cells in the wounded area shows the relative activity of migration. (D-F) Representative images of tube formation. Relative tube length and number of branches were quantified by measuring the cumulative tube length and branches. Scale bars = 200 μm. (G-K) The matrigel was premixed with siScr or sicZFP609, and was injected subcutaneously into *SIRT1*-Tg mice. (G) Bright field image of angiogenesis (upper lane) and representative images of CD31 (lower lane) and NG2 (I) immunostaining in matrigel plugs. Nuclei were stained with DAPI in blue. Scale bars = 100 μm. Angiogenesis in matrigel plugs was quantified by measuring CD31 (H) and NG2 positive area (J). (K) qRT-PCRs of CD31 mRNA, VEGFA mRNA and cZFP609 expression in matrigel plugs (n=5 mice per group). (A, C, E, F, H, J and K) Bar graphs show mean±SEM. Student's t-test: *P<0.05, **P<0.01 versus the corresponding control.

Hypoxia-induced angiogenesis is achieved through VEGFA-driven proliferation and migration of ECs 33. To verify the direct effect of cZFP609 on endothelial angiogenic functions, the expressing vector was transfected into ECs to overexpress cZFP609, and proliferation of ECs was determined by BrdU incorporation. We showed that overexpression of cZFP609 inhibited the proliferation of ECs in response to hypoxia (Figure 5A). BrdU incorporation was decreased to ~40% of that of the vehicle control. We then performed cell-wounding assay following overexpression of cZFP609 in ECs. The migration was attenuated in cZFP609-overexpressed ECs, consistent with reduced proliferative activity (Figure 5B-C). The tube formation assay is a preferred protocol to identify inhibitors or stimulators of the angiogenic activity of ECs *in vitro* 34. Using this protocol, we measured the effect of cZFP609 on the tubule formation in a quantifiable manner. Overexpression of cZFP609 resulted in shortened relative tube length (Figure 5D-E), accompanied with reduced relative number of branches in ECs transfected with cZFP609 vector compared with vehicle control (Figure 5F). To validate whether cZFP609 is a target to improve angiogenesis, the matrigel was premixed with siScr or sicZFP609, and was injected subcutaneously into *SIRT1*-Tg mice to knockdown of endogenous cZFP609 expression. We showed that the angiogenic dysfunction was reversed by knockdown of cZFP609 in *in vivo* matrigel plug assay (Figure 5G). Furthermore, the number of the capillaries invaded into the matrigel was significantly increased in the matrigel plugs with sicZFP609, accompanied with the density of NG2-positive cells (Figure 5G-J). qRT-PCR displayed that the expression level of CD31 and VEGFA mRNAs was elevated in the matrigel with sicZFP609 compared with the control (Figure 5K).These findings indicate that knockdown of cZFP609 improves endothelial angiogenic functions via promoting VEGFA expression in response to hypoxia.

### cZFP609 blocks hypoxia-induced HIF1α nuclear translocation in ECs

**Figure 6:**
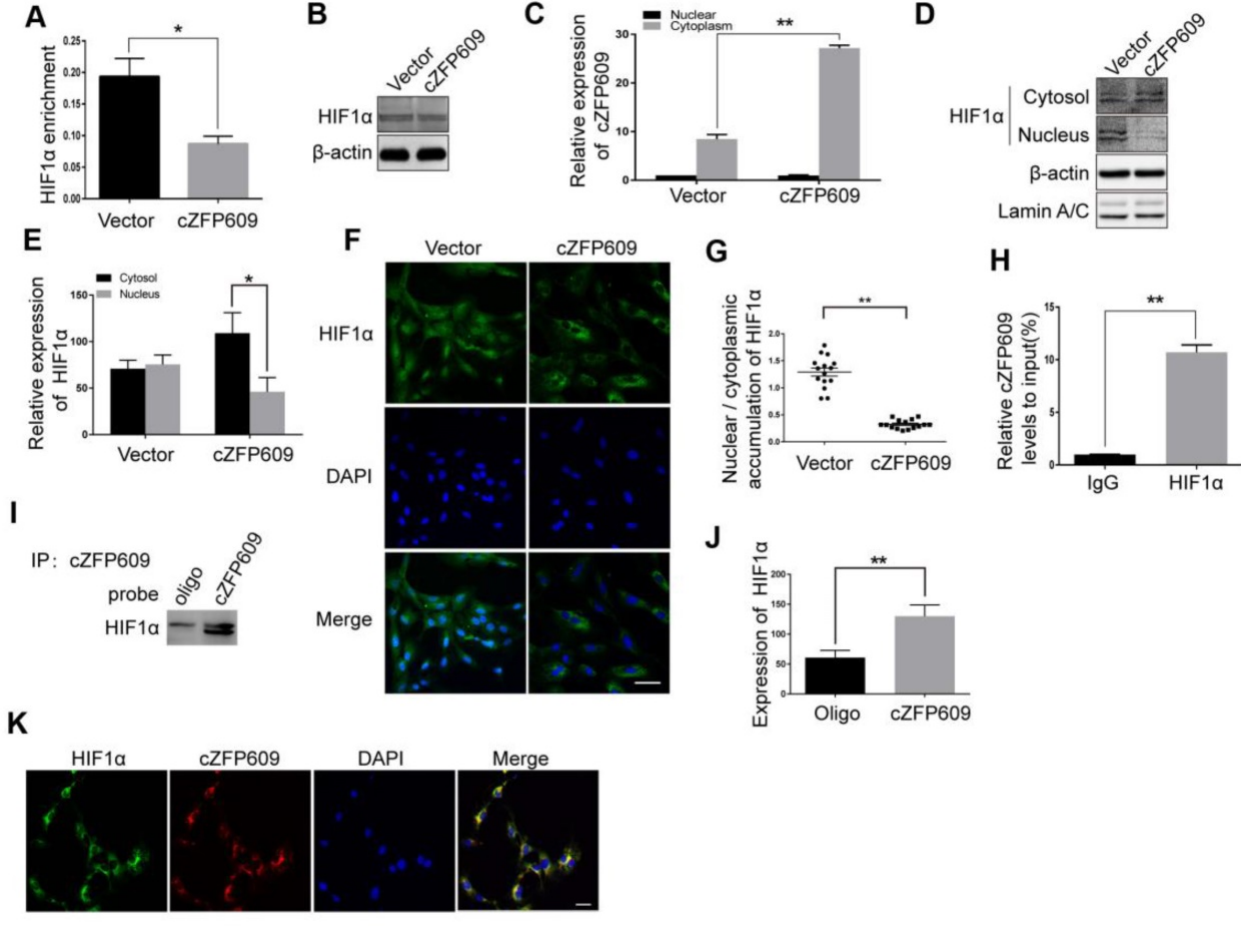
** cZFP609 interacts with and blocks HIF1α nuclear translocation in ECs.** The mouse ECs were transfected with vector or cZFP609 for 24 h and then incubated under hypoxia for 24 h. (A) ChIP assay for *VEGFA* gene promoter region in the ECs using HIF1α antibody. (B) Western blot analysis of HIF1α in the ECs. (C) qRT-PCRs for cZFP609 expression in the nucleus and cytoplasm of the ECs. (D and E) Western blot for HIF1α expression in the ECs. (F) Immunofluorescent confocal microscopy of HIF1α nuclear translocation in the ECs. Scale bars =100 μm. (G) The quantification of the nuclear-to-cytosol ratio of HIF1α protein in ECs (n=15). (H) RIP assay was performed using HIF1α antibodies in the ECs. qRT-PCR was used to detect pulled-down cZFP609. (I and J) The cytoplasm was extracted in ECs incubated under hypoxia for 24 h. RNA pull-down assay was performed using the probe. Western blot was used to validate the interactions between cZFP609 and HIF1α. (K) Confocal FISH images of colocalization between HIF1α and cZFP609 in the ECs. Scale bars=50 μm. Bar graphs show mean±SEM from 3 independent experiments (n=3). Student's t-test: *P<0.05, **P<0.01 versus the corresponding control.

HIF1α binding to the promoter region is essential for hypoxia-induced transcription of *VEGFA* gene. To validate the causal relationship between decrease in expression of VEGFA and nuclear HIF1α activity in ECs overexpressing cZFP609, we first performed ChIP assay using a HIF1α antibody to detect the binding activity between HIF1α and the promoter. We showed that overexpression of cZFP609 significantly decreased the binding of HIF1α to the promoter of *VEGFA* gene (Figure 6A) with unchanged expression of HIF1α in ECs (Figure 6B). We then isolated nuclear and cytosolic fractions from ECs to examine the distribution of cZFP609 in ECs using qRT-PCR. We found that cZFP609 was mainly localized in the cytoplasm, and significantly increased in the cZFP609-transfected ECs (Figure 6C). Using Western blot and immunofluorescence staining, we found that the distribution of HIF1α in the nuclei fraction decreased in ECs overexpressing cZFP609 in response to hypoxia (Figure 6D-E), and hypoxia-induced nuclear translocation of HIF1α disappeared under the same conditions (Figure 6F-G). To confirm that cZFP609 inhibits the nuclear translocation of HIF1α, we performed RIP and RNA pull-down assays, respectively. We demonstrated that cZFP609 was retrieved by HIF1α antibody (Figure 6H), and HIF1α protein was also retrieved by cZFP609 probe in ECs overexpressing cZFP609 (Figure 6I-J). In addition, the cZFP609 binding to HIF1α was further verified by fluorescence *in situ* hybridization (FISH) assay, which displayed the co-localization of cZFP609 and HIF1α in the cytoplasm of ECs (Figure 6K). Taken together, our data demonstrate that cZFP609, as a sponge, detains HIF1α in the cytoplasm, resulting in suppression of hypoxia-induced VEGFA expression.

### cZFP609 is negatively correlated with blood flow perfusion after ischemia

**Figure 7:**
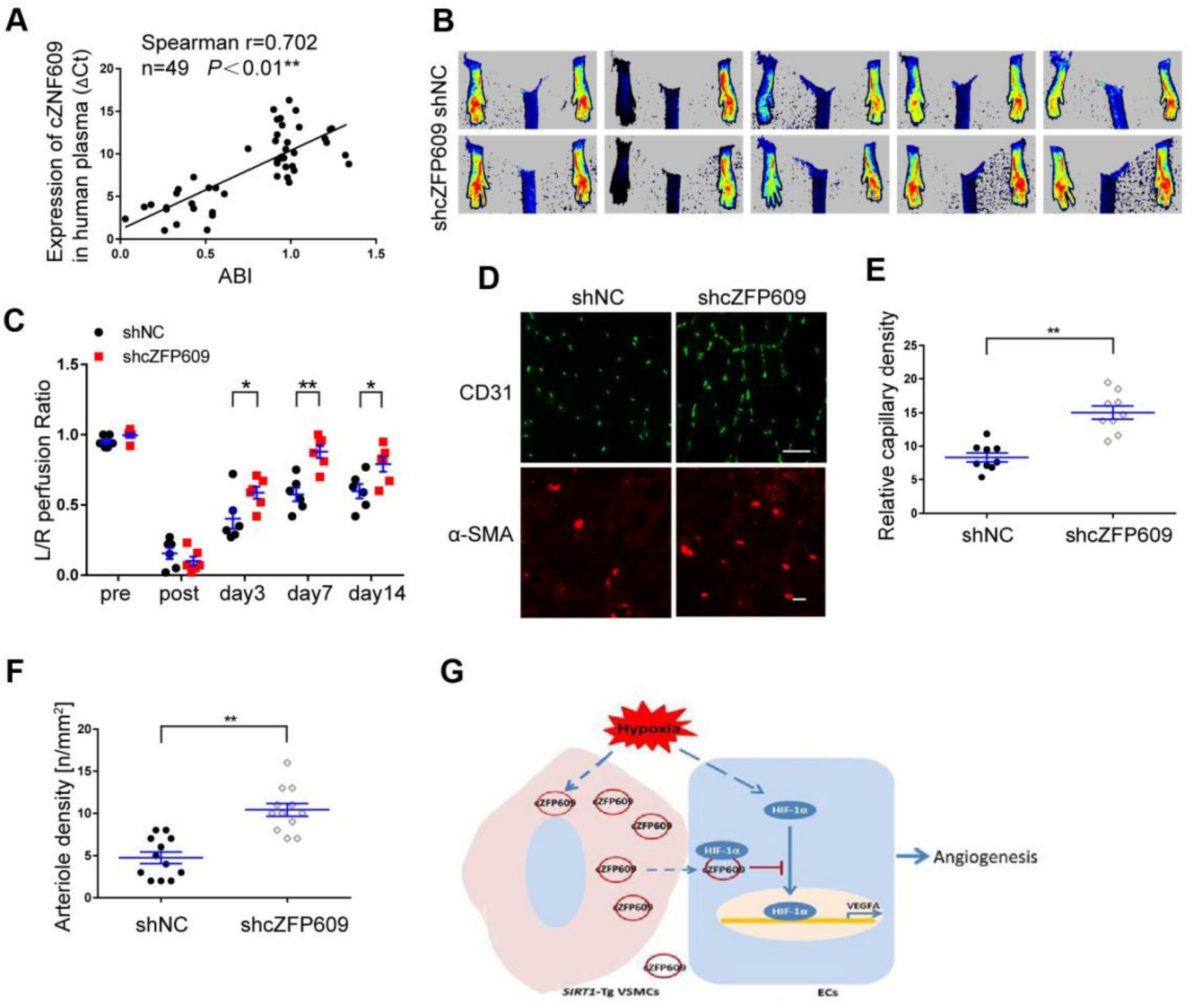
**cZFP609 negatively regulates blood flow perfusion.** (A) Plasma samples were collected from the patients with PAD (n = 19) and control subjects (n = 30). The plasma cZNF609 was determined by qPCR. ABI was calculated by measuring the ankle and the brachial systolic blood pressure using PeriFlux System 5000 (Perimed AB, Datavagen 9A, 175 43 Jarfalla, Sweden). Spearman's correlation analysis was performed. (B) Representative laser Doppler perfusion images at indicated time point after hindlimb ischemia. *SIRT1-*Tg mice treated with shcZFP609 or shNC (n= 6). (C) Laser Doppler perfusion at various time points was expressed as a ratio of flow between ischemic (L) and sham (R) limbs (L/R). (D) Immunofluorescence for CD31 (Scale bars = 100 μm) and α-SMA-positive cells (Scale bars = 20 μm) in the gastrocnemius tissue. (E) Immunostaining of CD31-positive cells represented the relative capillary density. (F) The density of arteriole was expressed as the quantity of arterioles per mm^2^. Data represent mean±SEM. Repeated Measures ANOVA or student's t-test: *P<0.05, **P<0.01 versus the corresponding control. (G) A working model for *SIRT1*-Tg VSMCs to impair angiogenesis via inhibition of HIF1α.

To explore the clinical value of cZNF609 in assessment of blood flow perfusion during tissue ischemia, we measured the plasma level of cZNF609 (cZFP609 in mouse or cZNF609 in human) in patients with atherosclerotic or diabetic lower extremity PAD (n=19). We showed that the plasma level of cZNF609 was significantly higher in the patients than that in normal individuals (n=30), and correlated with reduced ankle-brachial index (ABI), which reflected reduced blood flow in lower extremity (Figure 7A, Table S2). To explore whether inhibition of cZFP609 expression improves blood flow perfusion after ischemia, AAV9-shcZFP609 (1×10^12^ vg /mL) was injected into the gastrocnemius of *SIRT1-*Tg mice followed by the femoral artery ligation 21. We observed that knockdown of cZFP609 promoted early appearance of blood flow recovery (Figure 7B-C), and increased number of capillaries and arterioles in the ischemic tissues of *SIRT1*-Tg mice compared with the control (Figure 7D-F). These data indicate that plasma cZNF609 level can reflect the severity of lower extremity PAD in patients to some extent.

## Discussion

The results of the present study reveal that VSMC SIRT1 negatively regulates angiogenesis, resulting in a delayed blood flow recovery following hindlimb ischemia, similar to results from RSV administrate. Mechanically, the VSMCs-derived cZFP609 could be delivered to ECs by exosomes, and attenuated endothelial angiogenic function via blockade of HIF1α nuclear translocation and inhibition of VEGFA expression in response to hypoxia (Figure 7G). Higher cZFP609 expression was observed in the plasma, and associated with reduced ABI in the patients with atherosclerotic or diabetic lower extremity PAD. Thus, cZFP609 may be a novel biomarker and potential therapeutic target in treatment of ischemic diseases.

The restoration of hindlimb perfusion after ischemia is dependent on angiogenesis as well as capillary arterialization. VEGFA plays a central role in development and postnatal angiogenesis, and the source of the growth factor and the stimulus for its production include ischemic tissues, infiltrating monocytes/macrophages and the blood vessels themselves. The key factor regulating VEGFA production in ischemic tissues is HIF1α. HIF1α levels in turn are controlled in a highly complex manner by several regulators 35. The recent study showed that the activation of the endothelial NF-κB cascade can lead not only to local production of VEGF but also to accumulation of monocytes/macrophages due to increased expression of adhesion molecules leading to further increase in local accumulation of VEGFA 27. Furthermore, activated ECs induce the expression of PDGF-AA, PDGF-BB, and TGF- β in VSMCs, and the shear stress modulates VSMC migration, apoptosis, proliferation, and gene expressions in an EC-dependent manner 36, 37. According to a series of research results, activation of ECs and accumulation of monocytes/macrophages are considered the principal regulatory mechanisms for angiogenesis after ischemia. However, there is almost no attention to the regulating role of VSMCs on this process. In the present study, we first demonstrated that VSMCs actively reprogrammed the angiogenic functions of ECs using SIRT1-overexpressed VSMCs in response to hypoxia *in vivo* and *in vitro*. We showed that the blood flow recovery was delayed, and angiogenesis was impaired in *SIRT1*-Tg mice after ischemia. Our findings suggest that VSMCs may be independently associated with angiogenesis rather than not only involved in arteriogenesis.

The remarkable plasticity of the VSMCs, particularly their ability to change phenotype from the contractile to the synthetic in response to hypoxia, makes the proliferation possible through a HIF1α-dependent mechanism 38. VSMC phenotypic changes are typical for arteriogenesis 2. The activation of SIRT1 in VSMCs prevents neointimal formation, hypertension and atherosclerosis 11, 16, 39. We showed that overexpression of SIRT1 modulated VSMCs to be over-differentiated phenotype, which maintained higher expression of the contractile proteins and reduced synthetic markers in response to hypoxia, suggesting that SIRT1 might attenuate the phenotypic plasticity of VSMCs. The non-adaptive phenotype of VSMCs may result in generation of the abnormal signal component and lack of growth factors, and then disturb the angiogenic functions of ECs. It illustrates well the importance of balanced VSMC phenotype plasticity for normal vascular function.

SIRT1 is highly expressed in the vasculature during blood vessel growth. It's well known that SIRT1 controls the angiogenic activity of ECs via deacetylation of FoxO1 and uniquely regulates angiogenesis signaling 10. Loss of SIRT1 function blocks sprouting angiogenesis and branching morphogenesis of ECs with consequent down-regulation of genes involved in blood vessel development and vascular remodeling that is essential for postnatal blood vessel development. The inactivation of SIRT1 causes reduced vascular branching and density *in vivo* 26. However, other study revealed SIRT1 has negative effects on angiogenesis via deacetylating and inactivating HIF1α 7. Moreover, SIRT1 is transcriptionally downregulated during hypoxia to modulate cellular adaptation to hypoxia by targeting HIF1α 40. In the present study, we confirmed that overexpression of SIRT1 in VSMCs inhibited the angiogenic response *in vivo* and *in vitro* via exosome cZFP609-mediated intercellular communication, whatever the role of SIRT1 of ECs. Our findings provide evidence that VSMCs may reprogram angiogenesis, and targeting VSMCs may operate the balance between positive and negative mechanisms of angiogenesis in a cell-specific manner.

circRNAs are a novel class of non-coding RNAs that form a covalently closed continuous loop, and are conserved and stable 31. circRNAs are specifically expressed in a cell type or developmental stage, indicating that circRNAs may play important roles in many physiological and pathophysiological processes. It has been demonstrated that circRNAs regulate gene expression by acting as miRNA sponges, RNA-binding protein sequestering agents, or nuclear transcriptional regulators 41. The recent studies suggest that circRNAs are involved in angiogenesis or are regulated by hypoxia, such as cFoxo3, cZNF292, cZNF609, cITCH, cTHSD1 31, 32, 42. Silencing cZNF609 increases EC migration and tube formation, and protects EC against oxidative stress and hypoxia stress 32. In the present study, we showed that the expression of cZFP609 was significantly increased in *SIRT1*-Tg VSMCs and released via the exosomes. The exosome cZFP609 was delivered into ECs that expressed low cZFP609, and acted as HIF1α sequestering agent to block its nuclear translocation, led to a decreased HIF1α-driven *VEGFA* gene expression in ECs. VSMC-derived cZFP609 disturbed hypoxia-induced reprogramming of growth signals, and impaired endothelial angiogenic functions. Furthermore, knockdown of cZFP609 effectively improved the growth of capillaries and promoted blood flow recovery after ischemia *in vivo*. Additionally, the plasma level of cZNF609 was negatively correlated with the ankle-brachial index in patients with atherosclerotic or diabetic lower extremity PAD, suggesting the potential clinical value of cZNF609 in assessment of blood flow perfusion in tissue ischemia. Collectively, these findings provide strong support for cZFP609, as an intercellular messenger, to mediate VSMCs to reprogram angiogenesis of ECs, which may be an additional mechanism of HIF1α post-translation regulation.

However, several questions in understanding the production and function of cZFP609 in VSMC reprogramming angiogenesis still remain to be clarified. First, SIRT1 maintains the differentiated phenotype of VSMCs, whether cZFP609 attenuates the plasticity of VSMCs in response to hypoxia, and the mechanism underlying SIRT1 mediating cZFP609 formation, are unclear. Second, we have demonstrated that SIRT1 inhibits vascular inflammation via suppressing the activation of NF-κB and the expression of pro-inflammatory factors in VSMCs 24, 43, and however, whether SIRT1 inhibiting NF-κB signaling limits accumulation of monocytes/macrophages due to decreased expression of adhesion molecules further leading to decrease in local level of VEGFA, a crucial pathological process involved in angiogenesis after ischemia, thereby impairing angiogenesis after ischemia, also remains unknown. Third, a recent study demonstrated that cZFP609 can be translated into a protein in a splicing-dependent and cap-independent manner 44, further investigation is required to determine whether its translated protein is involved in regulation of angiogenesis.

In summary, our study, for the first time, demonstrate that VSMC cZFP609 reprograms ECs to attenuate angiogenesis after ischemia via inhibition of HIF1α activation in SIRT1-dependent manner. Our findings provide not only novel insight into the molecular mechanism and cellular regulatory network in angiogenesis after ischemia, but also new valuable indicator to assess the prognosis and clinical outcomes of ischemic diseases.

## Supplementary Material

Supplementary figures and tables.

## Abbreviations

AAV: adeno-associated virus
ABI: ankle-brachial index
CD31: cluster of differentiation 31
ChIP: chromatin immunoprecipitation
EC: endothelial cell
FISH: fluorescence *in situ* hybridization
HIF: hypoxia-inducible factor
HUVEC: human umbilical vein endothelial cell
PCR: polymerase chain reaction
PDGF: platelet-derived growth factor
RIP: RNA immunoprecipitationn assay
SIRT1: silent information regulator 1
*SIRT1*-Tg: *SIRT1*-transgenic
VEGF: vascular endothelial growth factor
VSMC: vascular smooth muscle cell
WT: wild-type
