# Supplementary material for: Smooth muscle SIRT1 reprograms endothelial cells to suppress angiogenesis after ischemia
Source: Theranostics. 2020 Jan 1;10(3):1197–212. doi: 10.7150/thno.39320 (PMC6956806; doi:10.7150/thno.39320)
Supplement: Supplementary file 1 — Supplementary figures and tables. [file thnov10p1197s1.pdf]

Supplementary Materials:

Supplemental Table 1. PCR primer sequence, siRNA sequence, FISH probe sequence and primers applied in PCR genotyping on mouse genomic DNA

Primer sequence		
Mouse PDGFB	Forward	ATGAAATGCTGAGCGACCACT
	Reverse	TCAGCCCCATCTTCATCTACGG
Mouse VEGFA	Forward	TATGGCTGGCTGGGTCACTA
	Reverse	GCTTTGTTCTGTCTTTCTTTGGTC
Mouse TUBULIN	Forward	ACAATGCCACCCTTTCAGTCCA
	Reverse	CACTAGATGGTTCAGGTCACCGTA
Mouse ZFP609	Forward	TCTCCAGGTTATGCACAGTCCA
	Reverse	TCTTTACTTTCCCTTCTACGCTCT
Mouse cZFP609	Forward	GGCCACTAAAGAAAGTCAAGTCTG
	Reverse	GGACATCTTAGAGTCAACGTCCC
Mouse circFoxo3	Forward	ACGGGTTGGATTTTAACTTTGACT
	Reverse	TCATTCTGAACGCGCATGAAGC
Mouse circZFP292	Forward	TGCTCTCCACTATCCTTTCC
	Reverse	CTCCAGCAAAGGTAAAGGGTC
Mouse circTHSD1	Forward	GCTGAAAACAGACTGCCCCT
	Reverse	CCGTTCTGTCGCTCAGTGCC
Mouse circITCH	Forward	AGACCCCAGAAGCCAAGGTCA
	Reverse	TCCTCTGATCCATTTGTGCTC
Mouse CD31	Forward	AGCTAGCAAGAAGCAGGAAGGACA
	Reverse	TAAGGTGGCGATGACCACTCCAAT
Mouse QKI	Forward	AACAACTTGAAGCAGAAACGGGAT
	Reverse	TTCACTTCTTCAACCGCTCT
Human cZNF609	Forward	CCTCCAGCCAGTTCCCTTG
	Reverse	GTTCTCAGACCTGCCACATTG
Human GAPDH	Forward	ATCTTCCAGGAGCGAGATCCC
	Reverse	TGAGTCCTTCCACGATACCAA
siRNA sequence		
Scrambled siRNA	Forward	UUCUCCGAACGUGUCACGUTT
	Reverse	ACGUGACACGUUCGGAGAATT
circZFP609 siRNA	Forward	GUCUGAAAAGCAAUGAUGUTT
	Reverse	ACAUCAUUGCUUUUCAGACTT

QKI siRNA	Forward	GAGAAUCCUUGGACCUAGATT
	Reverse	UCUAGGUCCAAGGAUUCUCTT
RNA pull down probe (biotin)		
Oligo		AAGAAAGTCAAGTCTGAAAAGCAA TGATGTTGTCCACTGG
Mouse cZFP609		CCAGTGGACAACATCATTGCTTTTC AGACTTGACTTTCTT
Oligo		ACGCAATTACATCATTCGGT
ZFP609 pre-mRNA1		AACAATACAGGCAAGGAGAT
ZFP609 pre-mRNA2		TTAACTTCTCCTACCACTGC
ZFP609 pre-mRNA3		GTAGCCACCAATAGAATCCT
ZFP609 pre-mRNA4		CATCTAGCAACAAGTGTCTT
Fish probe		
Mouse cZFP609-Cy3		CCAGTGGACAACATCATTGCTTTTC AGACTTGACTTTCTT
Chip primer		
Amplified site on genome		
Mouse pdgfb intron	Forward	CAGCCCTACCCATGCCCAA
9708～9746	Reverse	AACAGGGCTCCAGGGACCA
Mouse vegfa promoter	Forward	CACCCTGGCTTCAGTTCCC
–933～–888	Reverse	TGAGCCCTTGTCTGATCTGC
QKI RIP qPCR		
zfp609 Intron 1-1	Forward	AGTGGTTTTCCAATGGTAAAACTATG
	Reverse	CCCCAAGTCATAACGTATCACCT
zfp 609 Intron 1-2	Forward	TGATTCAGACAGGAGTACCAGC
	Reverse	ATGTCACACTGGACCTCGAGA
zfp 609 Intron 2-3	Forward	CACTGCCCATCAGAACTGCC
	Reverse	ATTACCAGCTGCTCTGACCTTG
zfp 609 Intron 2-4	Forward	TAACTTTGACATAAGCTTTGCCTC
	Reverse	TAGTAAGTTCTAGGCAATCCAGG
Primers applied in PCR genotyping on mouse genomic DNA		
Gene		
Human SIRT1	P1: CTTCAGGTCAAGGGATGGTAT	
	P2: GCGTGTCTATGTTCTGGGTAT	
Sirt1-LoxP	oIMR7909: GGTTGACTTAGGTCTTGTCTG	
	oIMR7912: CGTCCCTTGTAATGTTTCCC	

SM22a-Cre	oIMR7055: GGCCCAGGGGTTGTCAAAATAGTC<br>oIMR7056: CTCCTCCAGCTCCTCGTCATACTTC<br>oIMR7057: CGCCGCATAACCAGTGAAACAG

Supplemental Table 2 Comparison of baseline characteristics of patients with lower extremity PAD or control subjects

	ABI＜0.8<br>n=19	ABI＞0.9<br>n=30	*P*
Age (years), (mean ±SD)	63.17±7.49	63.89±9.89	0.771
Gender (male), n (%)	13(68.42)	16(53.33％)	0.295
Intermittent claudication or rest pain, n (%)	19(100)	0(0)	P<0.001**
The extremities with ulceration, n (%)	4(21.05)	0(0)	0.037*
The extremities with gangrene, n (%)	2(10.53）	0(0)	0.145
Current smoking, n (%)	7(36.84)	12(40.00)	0.825
Former smoker, n (%)	12(63.16)	16(53.33)	0.498
SBP (mmHg), (mean ±SD)	135.13±17.51	142.21±23.25	0.231
DBP (mmHg), (mean ±SD)	81.27±11.33	77.95±11.36	0.323
Hypertension, n (%)	16(84.21)	21(70.00)	0.432
Diabetes, n (%)	6(31.58)	10(33.33)	0.898
Hyperlipidaemia, n (%)	2(10.53)	4(13.33)	1.000
Prior stroke,	5(26.32)	9(30.00)	0.781
Chronic kidney disease	1(5.26）	2(6.67)	1.000
Carotid artery disease	3(15.79）	5(16.67)	1.000
Coronary heart disease	7(36.84)	11(36.67)	0.990
Heart failure	1(5.26)	2(6.67)	1.000

ABI, ankle-brachial index; PAD, peripheral artery disease

Supplemental Figure 1

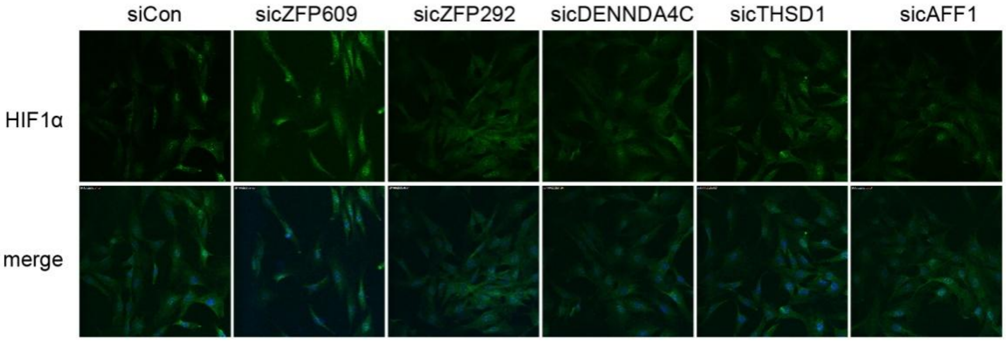

Figure S1 HIF1α nuclear translocation induced by hypoxia in ECs. ECs were treated with the exosomes from *SIRT1*-Tg VSMCs knocked down for these circRNAs under hypoxia.
